# KNOWLEDGE AND COMPLIANCE TO PRACTICE OF PREVENTIVE MEASURES TO COVID-19 AMONG NURSES IN A SELECTED TERTIARY HOSPITAL IN SOUTH-SOUTH, NIGERIA

**DOI:** 10.21010/Ajid.v16i2.6

**Published:** 2022-05-06

**Authors:** Odikpo Linda C., Ezike Okwudili Calistus, Onyia Evert O., Egbuniwe Michael Chiedu, Clementina I. Ilo, Obidife I. Helen, Clementina U. Nwankwo

**Affiliations:** 1 Department of Nursing Science, Nnamdi Azikwe University, Awka, Nigeria; 2 Federal Medical Centre, Asaba, Delta State, Nigeria; 3 Federal Medical Centre, Asaba, Delta State, Nigeria; 4 Department of Nursing Services, Nnamdi Azikwe University teaching hospital, Nnewi; 5 Department of Nursing Science, Nnamdi Azikwe University, Awka, Nigeria; 6 Department of Nursing Science, Nnamdi Azikwe University, Awka, Nigeria; 7 Department of Nursing Science, Nnamdi Azikwe University, Awka, Nigeria

**Keywords:** Knowledge, Compliance, preventive measures, Nurses, COVID-19

## Abstract

**Background:**

Healthcare providers have been at the Front line the response to the COVID-19 disease. Many of them have contracted the disease, and some of them already dead. This study assessed the knowledge, compliance with preventive measures and determined the relationship between knowledge and practice of preventive strategies to COVID-19 among nurses working in a selected hospital in South-South Nigeria.

**Materials and methods:**

A cross-sectional descriptive design guided the study. Census method guided the recruitment of all the 378 nurses in the hospital who met the study’s inclusion criteria.

**Results:**

Majority (360 [95.2%]) of the nurses had good knowledge of the preventive measures to COVID-19 and 311 (82.4%) of the nurses adhere strictly to practice of the preventive strategies to COVID-19. Educational level and years of experience are determinants of knowledge about preventive measures to COVID-19 (*p<0.05 respectively)* while knowledge, sex, level of education, years of experience, and unit of practice are determinants of compliance to preventive measures to COVID-19 among the nurses (p<0.001). Female nurses (p=0.012), RN/RM qualified nurses (p=0.037), nurses with more than five years of experience, and those in children ward (p=0.020) and maternity complex (p=0.003) significantly comply more to the preventive measures for COVID-19 as shown by their adjusted odds ratios.

**Conclusion:**

As knowledge of COVID-19 preventive strategies continues to increase among health workers, there is a need to translate this knowledge into adequate practice in order to minimise the hazardous effect of the pandemic on the health workers especially nurses.

## Introduction

Coronavirus disease 2019 (COVID-19) is an infectious and contagious disease caused by a newly discovered coronavirus and one of the biggest killers of 2020 (WHO, 2020; The Economist, 2020). The illness was first discovered in December 2019 in Wuhan, the capital of Hubei province in China and has since escalated to different parts of the world, leading to the ongoing 2019-2020 coronavirus pandemic (Hui *et al.*, 2020).

The virus primarily spreads among people during close contact, often via small droplets produced by coughing, sneezing, or talking (WHO, 2020). People, including health workers, may also become infected by touching a contaminated surface and then touching their eyes, nose, or mouth especially nurses who spend more time with patients than other professionals. The symptoms experienced by persons who contracted the COVID-19 virus include cough, fever, headache, chest pain including mild to moderate respiratory illness. Older people and those with underlying medical problems are more likely to develop severe illness (WHO, 2020).[U1] The best way to stem the tide of this deadly scourge on the human population is to get adequate information about the virus, its causes and how it spreads. More so, washing of hands or using an alcohol-based rub frequently and not touching one’s face, including wearing of personal protective equipment (PPE[M2]) by health workers, especially nurses, are right preventive measures against the disease (WHO, 2020). Everybody, especially nurses that are always in close contact with the patients, is expected to practice appropriate preventive strategies which are the most important interventions to prevent themselves from being infected by the coronavirus disease.

The significant occupational risks that increase the rate of transmission of COVID-19 infection among health workers, especially nurses, include late recognition or suspicion of COVID-19 in patients, working in a higher-risk department, longer duty hours, sub-optimal compliance to measures for prevention and control of infection such as effective handwashing and lack of or improper use of personal protective equipment (PPE). The WHO on 28th April 2020, raised awareness of health workers on the need for infection prevention and control (IPC[U3]) protocol which can aid the reduction of the number of deaths and injuries associated with work (WHO, 2020).

Several studies have revealed the fact that some Health care workers (HCWs’)[U4] have inadequate awareness of infection prevention practices (Wu *et al.*, 2020). Moreover, knowledge of disease can influence HCWs’ attitudes and practices, and[U5] incorrect attitudes cum practices of the preventive measures directly increase the risk of infection (McEachan *et al.*, 2016). With regards to practices of preventive measures to COVID-19, research evidence shows that it is associated with work experience, working time, and other factors. Non-frontline HCWs were less likely to maintain quarantine with family than frontline workers, especially nurses (Minghe *et al.*, 2020). Moreover, nurses must, in most cases, collect saliva samples from patients’ pharyngeal isthmus, and if they neglect their protection in this process, they may significantly increase the risk of the infection among themselves. Moreover, they are more vulnerable to the infection if they do not wash their hands carefully and frequently (Murthy *et al.*, 2020). Therefore, it is necessary to possess adequate knowledge and comply with the ideal protocol to prevent further contamination and infection (WHO, 2020) despite the barriers that might be present within the practice settings including inadequate PPE.

In Nigeria, however, there are lots of reports presently on the alarming level of infection and death of frontline health workers, including nurses. Poor compliance to COVID-19 preventive measures seems to be the prime cause of this increasing rate of COVID-19 infection among the health workers. The extent of what health workers know about the preventive measures seemingly determines the extent of their compliance with the measures. Therefore, this study assessed knowledge and compliance with the practice of preventive measures to COVID-19 among nurses in a selected hospital in South-South, Nigeria.

## Materials and Methods

The design of this study is a cross-sectional descriptive survey. We chose it as the most suitable design to assess the knowledge and evaluate compliance with infection prevention protocols for COVID-19 among Nurses in a selected hospital in Asaba, South-South Nigeria. Federal Medical Centre (FMC) Asaba was the setting for the study.

MC Asaba is a tertiary health institution located in Oshimili South Local Government Area, Delta State in South-South geopolitical zone of Nigeria. The study population consists of all registered nurses in the centre, which is a total of 466 (Nurses’ Annual Report, 2019). Recruitment of all the 278 nurses in the hospital who met the study’s inclusion criteria was through the census method.

The study instrument was a self-structured questionnaire validated by research experts consisting of a microbiologist, health educationist, and statistician for appropriateness of the content of the survey instrument. With the instrument, we conducted a pilot study using twenty randomly selected nurses at Okwe General Hospital in Asaba, by the test-retest method to ensure the reliability of the instrument. Data generated from the pilot study was tested using Cronbach alpha reliability method, and we obtained a reliability coefficient (r) of 0.870, which showed that the instrument was reliable. We collected, collated and analysed the main data using descriptive statistics which include frequency, percentages, means and standard deviations. The results are presented in tables. Chi-square and multivariate regression analysis guided test of association between practice and knowledge, and socio-demographic profiles and practice with knowledge. The Research Ethics Committee of a federal government-owned tertiary hospital in Asaba granted ethical approval for the study. An administrative permit was also obtained by the researchers from the appropriate authorities in the Nursing services department of the hospital before data collection. The researchers obtained informed consent from each respondent before data collection, and each participant had to first complete the consent form before completing the questionnaire. The ethical clearance approval for the study was obtained on the 1^st^ of June, 2020.

## Results

Table 1 shows the respondents’ socio-demographic characteristics. The mean age of the respondents is 41.4 ± 7.8, the majority (354 [93.7%]) are females, 286 (75.7%) are married, and 244(64.6%) had double qualifications (RN/RM). The modal years of experience fell within 6-10 years of experience, and the highest number (88 [23.3%]) of the respondents were in maternity complex.

**Table 1:** Socio-demographic characteristics of the respondents

Variable	Frequency	Percentage
**Age category (Years)**		
less than 30	120	31.7
31 – 40	188	49.7
41 – 50	52	13.6
51 and above	18	4.8
**Mean age (Years)**	41.4 ± 7.8	
**Sex**		
Male	24	6.3
Female	354	93.7
**Marital status**		
Married	286	75.7
Single	61	16.1
Widow	24	6.3
Divorced/separated	7	1.9
**Religion**		
Christian	369	98.9
Islam	4	1.1
Traditional	0	-
**Qualification**		
RN	4	1.1
RN/RM	244	64.6
B.SCN	112	29.6
MSN/PhD Nursing	18	4.8
**Years of experience**		
1 – 5	59	15.6
6 – 10	157	41.5
11 – 15	100	26.5
16 – above	62	16.4
**Unit**		
Medical wards	42	11.1
Surgical wards	44	11.6
Children ward	28	7.4
Clinics	82	21.7
Theatre	31	8.2
Isolation wards	23	6.1
Maternity complex	88	23.3
Emergency unit	40	10.6

Table 2 shows knowledge of the preventive strategies to COVID-19. Majority of the nurses know that constant washing of hands, physical distancing, coughing and sneezing into the elbow, wearing and disposing of PPE appropriately, staying at home when sick, decontamination of high touch surfaces before and after work are all preventive strategies to COVID-19. On the overall, 360(95.2%) have good knowledge of the practice of preventive measures to COVID-19.

**Table 2a:** Knowledge of preventive strategies to COVID-19 among the respondents

Questions		Yes (%)	No (%)
1.	Constant washing of hands is a preventive strategy to COVID-19	374 (98.9)	4 (1.1)
2.	Physical distancing is a preventive strategy to COVID-19	374 (98.9)	4 (1.1)
3.	Not touching one’s nose, mouth and eyes helps to prevent COVID 19	376 (99.5)	2 (0.5)
4.	Coughing and sneezing into the armpit or a tissue paper and disposing of it immediately prevent the spread of COVID-19	362 (95.8)	16 (4.2)
5.	Wearing and disposing of already used PPEs prevent COVID-19	354 (93.7)	24 (6.3)
6.	Staying at home when sick prevents COVID-19	272 (72.0)	106 (28.0)
7.	Staying off work and self-isolating if one has any COVID-19 symptoms prevent the disease	319 (84.4)	59 (15.6)
8.	Decontamination of high touch surfaces before and after work prevents COVID-19	370 (97.9)	8 (2.1)

**Table 2b:** Knowledge score of the respondents on preventive strategies to COVID-19

Knowledge Scores	Frequency	Percentage
Mean score 7.4 ± 0.92		
6 – 8 (Good)	360	95.2
4 – 5 (Fair)	16	4.2
≤ 3 (Poor)	2	0.5

Table 3 shows the result of compliance with the preventive measures to COVID-19 among the respondents. Majority of the nurses practice the preventive measures to COVID-19 except for the practice of adequate physical distancing between them and the patients. 171 (45.2%) of the nurses reported not practising physical distance between them and the patients and 129 (34.1%) of them not wearing PPE always. On the overall, 311(82.4%) nurses adhere strictly to good practice of the preventive strategies to COVID-19.

**Table 3a:** Compliance to the preventive strategies to COVID-19 among the respondents

Variables		Always	Most times	Occasionally	Rarely
1.	Practice social distancing to prevent and reduce risk to COVID-19	66 (17.5)	233 (61.6)	79 (20.9)	0 (0.0)
2.	Practice handwashing with soap to prevent and reduce risk to COVID-19	240 (63.5)	128 (33.9)	10 (2.6)	0 (0.0)
3.	Avoid touching the nose, the mouth and the eyes to prevent and reduce risk to COVID-19	181 (47.9)	131 (34.7)	62 (16.4)	4 (1.1)
4.	Cough and sneeze into the elbow or tissue and disposed immediately to prevent and reduce risk to COVID-19	179 (47.4)	118 (31.2)	59 (15.6)	22 (5.8)
5.	Wear PPE always as a preventive measure to COVID-19	111 (29.4)	129 (34.1)	94 (24.9)	44 (11.6)
6.	Dispose of PPE properly to prevent and reduce risk to COVID-19	252 (66.7)	66 (17.5)	42 (11.1)	18 (4.8)
7.	Follow all the PPE’s protocol as approved by WHO to prevent and reduce risk to COVID-19	205 (54.2)	83 (22.0)	78 (20.6)	12 (3.2)
8.	When not at work, stay home to prevent and reduce risk to COVID-19	205 (54.2)	119 (31.5)	50 (13.2)	4 (1.1)
9.	Stay off work and self-isolate if one has any COVID-19 symptoms to prevent and reduce risk to colleagues and other patients	174 (46.0)	130 (34.4)	34 (9.0)	40 (10.6)
10.	Decontaminate high touch surfaces before and after work to prevent and reduce risk to COVID-19	232 (61.4)	72 (19.0)	62 (16.4)	12 (3.2)
11.	Practice hand hygiene immediately one touches patient body fluid accidentally to prevent and reduce risk to COVID-19	334 (88.4)	34 (9.0)	10 (2.6)	0 (0.0)
12.	Maintain adequate distance in the care of patients to prevent and reduce risk to COVID-19	144 (38.1)	171 (45.2)	55 (14.6)	8 (2.1)

**Table 3b:** Practice of the preventive strategies to COVID-19

Practice	Frequency	Percentage
Good practice	311	82.4
Poor practice	67	17.6

Table 4a shows the level of education, years of experience are determinants of knowledge about preventive measures to COVID-19 (*p<0.05respectively)*; while knowledge, sex, level of education, years of experience, and unit of practice are determinants of compliance to preventive measures to COVID-19 among the nurses as most of the p-values were p**<**0.001

**Table 4a:** Socio-demographic determinants of good knowledge and practice of COVID-19 preventive strategies

Variables	Knowledge		*X^2^* Test	*p-value*	Practice		*X^2^* Test	*p-value*
	**Adequate (376)**	**Inadequate (2)**			**Good (311)**	**Poor (67)**		
**Sex**								
Male	24 (6.4)	0 (0.0)	0.14	0.712	15 (4.8)	9 (13.4)	6.87	0.009*
Female	352 (93.6)	2 (100)			296 (95.2)	58 (86.6)		
**Knowledge**								
Adequate	-	-	-	-	348 (92.6)	28 (7.4)	23.3	<0.001*
Poor					0 (0.0)	2 (100.0)		
**Level of education**								
RN	3 (0.8)	1 (50.0)			2 (0.6)	2 (3.0)		
RN/RM	244 (64.9)	0 (0.0)	47.2	<0.001*	218 (70.1)	26 (38.8)	37.5	<0.001*
B.SCN	111 (29.5)	1 (50.0)			73 (23.5)	39 (58.2)		
MSN/PhD Nursing	18 (4.8)	0 (0.0)			18 (5.8)	0 (0.0)		
**Years of experience**								
1 – 5	57 (15.2)	2 (100)			32 (10.3)	27 (40.3)		
6 – 10	157 (41.5)	0 (0.0)	10.9	0.012*	136 (43.7)	21 (31.3)	38.1	<0.001*
11 – 15	100 (26.5)	0 (0.0)			87 (28.0)	13 (19.4)		
16 – above	62 (16.5)	0 (0.0)			56 (18.0)	6 (9.0)		
**Unit**								
Medical wards	42 (11.2)	0 (0)			40 (12.9)	2 (3.0)		
Surgical wards	44 (11.7)	0 (0.0)			44 (14.1)	0 (0.0)		
Children ward	27 (7.2)	1 (50.0)			25 (8.0)	3 (4.5)		
Clinics	82 (21.8)	0 (0.0)	6.93	0.436	63 (20.3)	19 (28.4)	50.7	<0.001*
Theatre	31 (8.2)	0 (0.0)			31 (10.0)	0 (0.0)		
Isolation wards	23 (6.1)	0 (0.0)			23 (7.4)	0 (0.0)		
Maternity complex	87 (23.1)	1 (50.0)			59 (19.0)	29 (43.3)		
Emergency unit	40 (10.6)	0 (0.0)			26 (8.4)	14 (20.9)		

* significant

Table 4b shows multivariate analysis on significant socio-demographic determinants of good knowledge and practice of COVID-19 preventive strategies. Nurses with BNSc had statistically significant good knowledge of the preventive measures to COVID-19 (*p*=*0.018)*; they are 37 times more knowledgeable about the preventive measures compared to those who had double diploma certificate (RN/RM) (AOR= 37.0, CI= 1.84-742.9). For the practice, female nurses (p=0.012), RN/RM qualified nurses (p=0.037), the nurses with more than five years of experience, those in children ward (p=0.020), maternity complex (p=0.003) and emergency unit significantly comply more to the preventive measures for COVID-19. From the adjusted odds ratios, compliance to preventive measured increased as years of experience increased. Those who had RN/RM were approximately eight times more likely to comply with the infection prevention protocol compared to those who had only RN (AOR=8.4); while those who had BNSc were only approximately two times more likely to comply with infection prevention protocols compared with those who had RN only (AOR = 1.9).

**Table 4b:** Multivariate analysis on significant socio-demographic determinants of good knowledge and practice of COVID-19 preventive strategies

Variables	KNOWLEDGE		Confidence interval	PRACTICE		Confidence interval
	*p*-value	AOR		*p*-value	AOR	
**Sex**						
Male	-	-	-	-	-	-
Female				0.012*	3.1	1.28 – 7.33
**Knowledge**						
Adequate	-	-	-	0.999	-	-
Poor						
**Level of education**						
RN	-	-	-	-	-	-
RN/RM	0.994	-	-	0.037*	8.4	1.13 – 62.1
BNSc	0.018*	37.0	1.84 – 742.9	0.539	1.9	0.25 – 13 8
MSN/PhD Nursing	0.998	-	-	0.998	-	-
**Years of experience**						
1 – 5	-			-	-	-
6 – 10	0.996			<0.001*	5.4	2.75 – 10.9
11 – 15	0.996	-	-	<0.001*	5.6	2.60 – 12.3
16 – above	0.997			<0.001*	7.9	2.9 – 21.1
**Unit**						
Medical wards				-	-	-
Surgical wards				0.998	-	-
Children ward				0.356	0.42	0.65 – 2.67
Clinics		-	-	0.020*	0.17	0.37 – 0.750
Theatre				0.998	-	-
Isolation wards				0.998	-	-
Maternity complex				0.003*	0.10	0.23 – 0.450
Emergency unit				0.003*	0.93	0.019 – 0.443

* significant

## Discussions

Result on knowledge of the preventive strategies to COVID-19 among nurses in the selected tertiary hospital in South-South region of Nigeria shows that[U6] the majority of the nurses know[U7] that constant washing of hands, physical distancing, coughing and sneezing into the elbow, wearing and disposing of PPE, staying at home when sick, decontamination of high touch surfaces before and after work are all preventive strategies to COVID-19. On the overall, the nurses have good knowledge of the preventive measures to COVID-19 and these preventive strategies were identified by WHO (2020) and African Field Epidemiology Network (2020) as the standard preventive measures to COVID-19 globally. This finding is in agreement with the result from Tendongfor *et al*. (2020), who reported in their study that the participants were aware that cleaning and disinfecting the environment could help prevent the infection. The use of face masks and hand sanitiser are also preventive strategies for COVID-19. Ronald *et al*. (2020) and Odikpo *et al*. (2021) reported similar findings in their study, which result revealed sufficient knowledge towards preventive strategies to COVID-19. Contrarily, Wu [U8]*et al*. (2020) in their study reported inadequate awareness of COVID-19 infection prevention practices among health workers. Yonas *et al*. (2020) also discovered a high prevalence of poor knowledge to preventive strategies to COVID-19, although among chronic kidney patients in Northwest Ethiopia.

On compliance to the preventive strategies to COVID-19 among the respondents, majority of the nurses complied except for the practice of adequate physical distancing between them and the patients (171 [45.2%), and the wearing of PPE (129 [34.1%). On the overall, 311(82.4%) nurses adhere strictly to good practice of the preventive strategies to COVID-19 in FMC Asaba. This finding resembles the report by Ronald *et al*. (2020), where their result revealed widespread acceptable practices toward the prevention of COVID-19. In a similar study although among the Chinese residents, Zhong *et al*. (2020) discovered appropriate practices toward COVID-19 among the Chinese residents which are invariably good compliance to the preventive strategies to COVID-19 as identified in our study. Another study by Chen *et al*. (2020) on practice about COVID-19 among residents in Anhui Province also revealed that the residents in Anhui province have good preventive practices about COVID-19. Similary, Muhammad *et al*. (2020) discovered that health workers in Pakistan have adequate knowledge, yet, there exist some aspects where gaps in knowledge and practice were prominent. Despite all these supportive studies, Yonas *et al*. (2020) discovered a high prevalence of poor practices to preventive strategies to COVID-19; although among chronic kidney patients in Northwest Ethiopia. The dissimilarity in Jonas *et al*. (2020) report might. be as a result of participants’ characteristics.

In a similar study on compliance with standard precautionary measures among health workers, Tariku *et al*. (2017) reported a finding of very low or inadequate practice. In the same vein, Sara *et al*. (2020) reported[U9] poor compliance to standard precautions as a result of some barriers identified by the majority of the health workers as a supported workforce that receives insufficient in-service training (Oleribe *et al.*, 2019). Hence the need for significant investments in the training of health workers especially nurses; and provision of resources urgently required to ensure healthcare facilities have the supplies and knowledge necessary for COVID-19 infection prevention and control activities. The result also revealed non-compliance to appropriate physical distance between the nurses and the patients, which can expose the nurses and other health workers as well to the disease. Therefore, the need to maintain at least a distance of one meter between the nurses and the patients and also between the patients in the hospital wards as stipulated by WHO (2020) is necessary to minimise the escalation of COVID-19 among health workers as well as other asymptomatic patients in the hospital wards.[U10]

Socio-demographic determinants of good knowledge and practice of COVID-19 preventive strategies shows that level of education and years of experience are determinants of knowledge about preventive measures to COVID-19 (*p<0.05respectively)*. Similarly, knowledge, sex, level of education, years of experience, and unit of practice are determinants of compliance to preventive measures to COVID-19 among the nurses as most of the p-values were < 0.001. The result was similar to findings by Valdivia *et al*. (2020)[U11] in which the result of logistic regression showed that knowledge correlated highly with education (p=0.031). This finding is contrary to the result by Ronald *et al*. (2020) who reported that factors associated with knowledge of preventive measures to COVID-19 include age of more than 40 years (aOR: 0.3; 95% CI: 0.1–1.0; *p* = 0.047) and news media (aOR: 4.8; 95% CI: 1.4–17.0; *p* = 0.015) while factors relating to good practices include age 40 years or beyond (aOR: 48.4; 95% CI: 3.1–742.9; *p* = 0.005) and acquisition of a diploma (aOR: 18.4; 95% CI: 1–322.9; *p* = 0.046). The result was also different from the present study as sex, years of experience, and unit of practice are determinants of compliance with preventive measures to COVID-19.

Multivariate analysis was carried out to determine the significant socio-demographic determinants of good knowledge and the practice of COVID-19 preventive strategies[U12]. Result showed nurses with BNSc had statistically significant good knowledge of the preventive measures to COVID-19 (*p*=*0.018)*; they are 37 times more knowledgeable about the preventive measures compared to those who had a double diploma certificate (RN/RM) (AOR= 37.0, CI= 1.84-742.9). For the practice, female nurses (p=0.012), RN/RM qualified nurses (p=0.037), the nurses with more than five years of experience, those in children ward (p=0.020), maternity complex (p=0.003) and emergency unit significantly comply more to the preventive measures for COVID-19. From the adjusted odds ratios, compliance with the preventive measure increased as years of experience increased. Those who had RN/RM were approximately eight times more likely to comply with the infection prevention protocol compared to those who had only RN (AOR=8.4); while those who had BNSc were only approximately two times more likely to comply with infection prevention protocols compared with those who had RN only (AOR= 1.9). From the foregoing, the level of education had a lot to do with the compliance of the nurses in FMC Asaba to the preventive measures to COVID-19. In contrast, Valdivia *et al*. (2020) and Ronald *et al*. (2020) identified sex (female nurses) and units as the determinants of compliance with preventive strategies to COVID-19.

This study is of serious implication to the Nigeria health care system, as no health care institution can thrive without health workers, especially nurses. Therefore, there is need for stakeholders from the ministry of health to make sure there are adequate equipment and supplies especially PPEs to be utilised by the health workers to prevent infection of the staff in various health care institutions. They should also activate the monitoring team in each institution of health to make sure that every health worker complies with the preventive protocols to COVID-19 as stipulated by WHO.

The limitation of the study includes not being able to use more than one hospital as a result of the pandemic and interstate travel ban by the government to curtail the spread of the COVID-19 virus. Secondly, researchers could not reach all the nurses in the hospital for the study as some were sick, quarantined due to the pandemic and as a result, could not participate in the study. From the above limitations, the generalisation of the study to entire South-South Nigeria may not be feasible

### Declaration of interest:

The authors declare there is no conflict of interest of any form with regards to this study.

### Funding sources:

There was no external source of funding received for this study from any funding institutions or donor.

### Ethical approval:

Ethical approval was obtained on 1^st^ of June, 2020 from **t**he Research Ethics Committee of a federal government-owned tertiary hospital in Asaba. An administrative permit was also obtained by the researchers from the appropriate authorities in the Nursing services department of the hospital before data collection.

**List of Abbreviations:**
COVID-19: Coronavirus disease 2019
PPE: Personal protective equipment
IPC: Infection prevention and control
HCW’s: Healthcare workers
WHO: World Health Organisation
